# Spirituality, Quality of Life, and Health: A Japanese Cross-Sectional Study

**DOI:** 10.3390/ejihpe14030050

**Published:** 2024-03-21

**Authors:** Takeshi Yoshizawa, Abdelrahman M. Makram, Randa Elsheikh, Sadako Nakamura, Engy Mohamed Makram, Kazumi Kubota, Nguyen Tien Huy, Kazuhiko Moji

**Affiliations:** 1 Department of Food Science and Graduate School of Human Life Sciences, Faculty of Human Life, Jumonji University, Saitama 352-8510, Japan; nacsacj@jumonji-u.ac.jp; 2 School of Public Health, Imperial College London, London SW7 2BX, UK; abdelrahman.elsayid21@imperial.ac.uk; 3 Department of Anesthesia and Intensive Care Medicine, October 6 University, Giza 12585, Egypt; 4 Deanery of Biomedical Sciences at Edinburgh Medical School, University of Edinburgh, Edinburgh EH8 9YL, UK; r.elsheikh@sms.ed.ac.uk; 5 Department of Plastic and Reconstructive Surgery, October 6 University, Giza 12585, Egypt; 6 College of Medicine, Misr University of Science and Technology, Giza 16878, Egypt; engymakram1704@gmail.com; 7 Department of Healthcare Information Management, The University of Tokyo Hospital, Tokyo 113-8655, Japan; kkubota@m.u-tokyo.ac.jp; 8 Institute of Research and Development, Duy Tan University, Da Nang 50000, Vietnam; nguyentienhuy4@duytan.edu.vn; 9 School of Medicine and Pharmacy, Duy Tan University, Da Nang 50000, Vietnam; 10 School of Tropical Medicine and Global Health, Nagasaki University, 1-12-4 Sakamoto, Nagasaki 852-8523, Japan

**Keywords:** spirituality, health, quality of life

## Abstract

Background: Current reports suggest a positive association between spirituality and quality of life (QoL) in elders. While most studies are qualitative studies and there has been little validation in quantitative studies using scales to measure spirituality. Hence, we aimed to study the effect of spirituality on mental health and QoL in older people residing in Kumejima Town in Japan. Methods: An interview-based survey was conducted between September 2010 and 2011 on residents of Kumejima Town aged 65 years or older. This survey-based study employed the Spirituality Health Scale for the Elderly (SP Health Scale) alongside assessments of basic attributes (e.g., age, sex); physical, mental, social health, spirituality, and QoL. We conducted a causal structure model to explore causal relationships between these factors. Results: Our study included 338 participants, including 72.5% female with an average age and standard deviation of 77.2 ± 6.4 years. Our analysis revealed a significant association between spiritual health and QoL even after accounting for the impact of physical and mental health, which challenged the conventional belief that QoL inevitably diminishes with age and declining health. These results suggest that enhancing spirituality may offer a means to prevent declines in QoL, fostering a positive outlook on life as individuals age. Conclusion: Our study suggests that improving spiritual health can enhance QoL, even in the presence of health challenges and aging. This novel perspective opens doors to redefining health as a state that coexists with illness, with spirituality serving as an integral component. A shift in our understanding of health that prioritizes spirituality, could benefit people of all ages, offering a more holistic approach to well-being that aligns with new medical technologies and evolving perceptions of health.

## 1. Introduction

The 1946 Constitution of the World Health Organization (WHO) was the first widely known document to include physical, mental, and social well-being in the definition of health [1]. Since then, many specialists have started wondering whether this definition of health is sufficient to provide humans with better lives [2]. This has led the WHO to expand the definition into specific pieces. For example, the Ottawa Charter of 1986 included the definition of health promotion to allow people to exercise their autonomy in their everyday decision-making (for example, what to eat, drink, or consume).

The Ottawa Charter also introduced health as a resource for living [3], which led some researchers to develop health metrics such as productivity-adjusted life-years [4]. Nevertheless, to this day, public health researchers are seeking to incorporate different angles of many problems in the definition of health [5].

This brought the idea of integrating spirituality into the definition of health when the representatives of Bahrain, Libya, and Sri Lanka proposed this to the WHO Executive Board in the WHO Fifty-second World Health Assembly of 1998. The common argument was that humans are not only a body (a chemical and physical form) but are also minds and souls. Moreover, since the definition of spirituality differs from country to country, it has been difficult to incorporate spirituality into the definition since it was first proposed in 1984. They also emphasized that religion should not be confused with spirituality [6].

In their systematic review conducted to define spirituality, de Brito Sena et al. summarized that spirituality includes 24 dimensions, most notably a connection/relation to something or someone, a meaning/purpose of life, believing in something divine or with limitless power like a God, leading an immaterial life, or having good community relationships [7]. Other domains are also crucial to understanding how the mind of a human shapes their experiences, even in the healthcare setting [7,8].

In this regard, we found that spirituality and spiritual health are not different from one another. For example, spirituality may be defined from the view of an individual by saying that a person who finds meaning, purpose, and fulfillment in life—often via relationships with others, themselves, nature, a higher power, or their belief system—is in a condition of spiritual health. It includes things like morality, ethics, values, and the pursuit of transcendence or inner peace.

Moreover, to link spirituality to health, we should acknowledge that suffering without meaning is the ultimate doom of our race [9]. For example, a patient suffering from a chronic illness may question their reality and fate. They may even become reluctant to receive their prescribed medicines [10]. Similarly, some studies have found that people who practice spiritual activities may live longer [11], are less stressed [12], cope better with pain [13,14,15], and have a better quality of life (QoL) [16]. 

However, the notion that QoL declines as death approaches focuses entirely on the biological aspects of human beings. For example, previous studies with older people have demonstrated that QoL also relates to frailty, mental health, social factors, psychological elements, and the surrounding environment. This shows that physical health and QoL do not necessarily fit into a linear relationship in older people [17,18,19,20,21]. Furthermore, research on aging has shown that the degree of spirituality is more potent in older people who are physically frail, suggesting that even if physical health declines with old age, spirituality can provide life satisfaction and better QoL [22,23]. 

Unfortunately, however, research on spirituality in Japan is dominated by qualitative studies, and there has been little validation in quantitative studies using scales to measure spirituality. This study was based on the hypothesis that spirituality should be included as the fourth health dimension in the WHO definition of health, that is, physical, mental, and social health, as it influences the QoL of older people. We, therefore, aimed to determine the relationship between spiritual health, mental health, and quality of life of old people.

## 2. Materials and Methods

### 2.1. Study Design

The reporting of this survey study was checked against the Consensus-Based Checklist for Reporting of Survey Studies (CROSS) [24], and the checklist is provided in Supplementary Table S1. 

### 2.2. Ethical Considerations

This study was conducted under approval number 107 of the Medical Ethics Review Committee of Kagawa Nutrition University. 

The objectives and content of the interview survey were explained in writing to the Kumejima Town civil workers, and their consent was obtained. The Kumejima civil workers explained the purpose of the study to the ward leaders of each village and the leaders of the Fureai Salons and obtained their consent. The Kumejima Town civil workers also explained the purpose of the research to the individual survey targets in advance, and only those who gave their consent were surveyed. The purpose and content of the interviews were explained verbally to the subjects themselves, and their consent was confirmed by asking them to sign a consent form. The subjects were promised that the content of the survey form would not be used for any purpose other than this research, that they would remain strictly anonymous, and that their privacy would be respected in the Ph.D. dissertation and the journal article.

### 2.3. Study Setting

**Figure 1:**
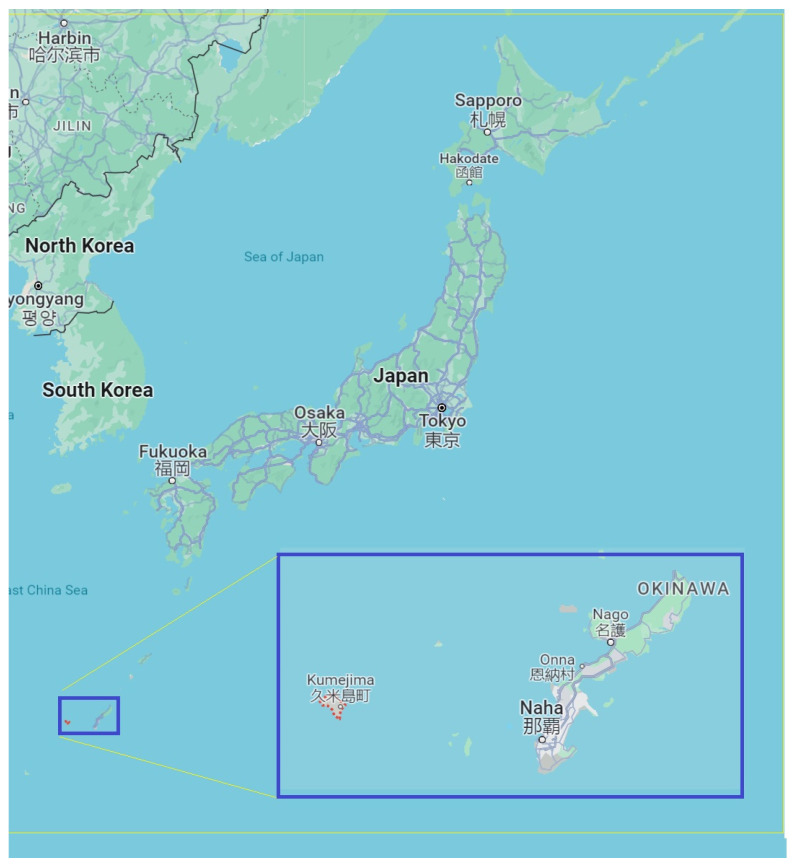
A map to illustrate the geography of the island with respect to Japan.

This study was conducted on people aged 65 years or older, who did not require nursing care, and who lived in Kumejima-cho, Okinawa Prefecture. Kumejima-cho, the surveyed area, is located on the East China Sea, 100 km west of Naha City on the main island of Okinawa, and is an island region with a circumference of approximately 48 km and an area of 63.65 km^2^ (Figure 1). There are three to five daily air flights from Okinawa Naha Airport, which take approximately 25 min. There are one to two boat services a day, which take about three hours. 

In 2020, Kumejima Town had a population of 7192, slightly more male residents, a population density of 113.0/km^2^, and a −1.5% annual change in population [25]. In Kumejima Town, the number of households with elderly persons aged 65 or older is 809, of which 311 are elderly couple-only households and 310 are elderly single-person households. The population aged 65 and over in Kumejima Town is 1685, with an aging rate of 20.1% (2010). This is slightly higher than the rate for Okinawa Prefecture as a whole (17.5% in 2009) and the national average (22.7% in 2009) [26]. 

### 2.4. Survey Development and Conduction

The survey was conducted in September 2010 and 2011 in an interview format (face-to-face) and using a collective method, which is surveying several participants at a time.

Our survey consisted of a total of 23 questions—further subdivided into multiple items—covering basic attributes (for example, age and sex); physical, mental, and social health; spirituality; and quality of life. The collected data did not include any information that could identify individual participants. 

We had ten questions about physical health, covering possible illnesses, sleeping habits, and eating habits. The mental health items were three and were about “subjective sense of health”, “subjective sense of satisfaction”, and “sense of stress”. The social health also included three items which were about the “frequency of going out”, “degree of neighborhood interaction”, and “role in family and community”. 

The Spirituality Health Scale for the Elderly (hereafter referred to as the SP Health Scale) [27] was selected as the scale to measure spirituality since it was developed to measure the spirituality of older people and has been tested for reliability and validity, considering the socio-cultural context of Japan. The original survey was in Japanese and was already tested and validated before. It consists of six sub-concepts: “meaning and purpose of living”, which aims at understanding individuals’ sense of meaning and purpose of life, “attitudes toward death and dying”, which aims at understanding individuals’ beliefs, perspectives, and emotions toward death, “self-transcendence”, which encompasses individuals’ sense of unity and interconnectedness with something greater or higher, “accordance with others”, which relates to one’s ability to establish harmonious relationships with others, “spiritual support”, referring to the practices used to nurture one’s spirituality, and “harmony with nature”, which focuses on individual’s relationship with nature. Each sub-concept consists of three questions, making a total of 18 items. For spirituality, a score from 1 to 5 was given, with higher scores reflecting stronger spiritual health [27]. 

Questions on QoL were developed based on Lawton’s revised Philadelphia Geriatric Center Morale Scale, which consists of 17 items measuring agitation, attitude toward own aging, and lonely dissatisfaction [28]. 

### 2.5. Data Analysis

**Figure 2:**
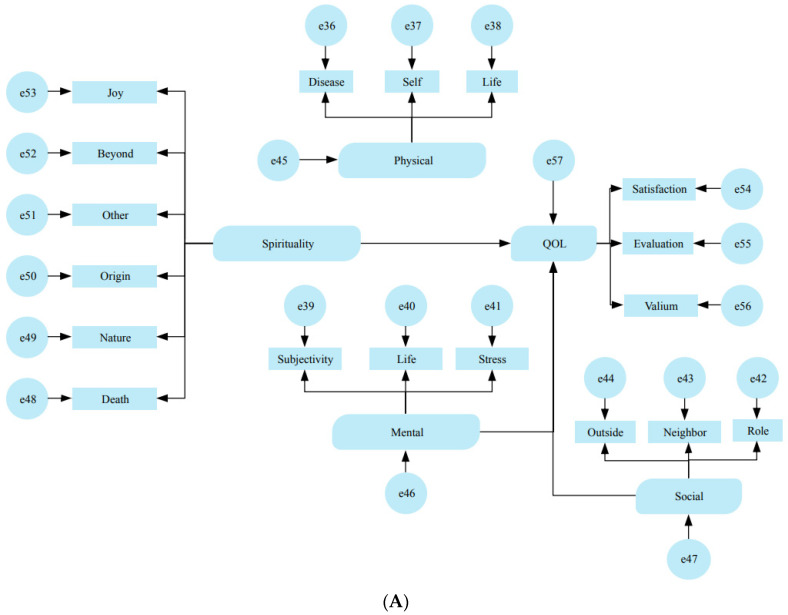

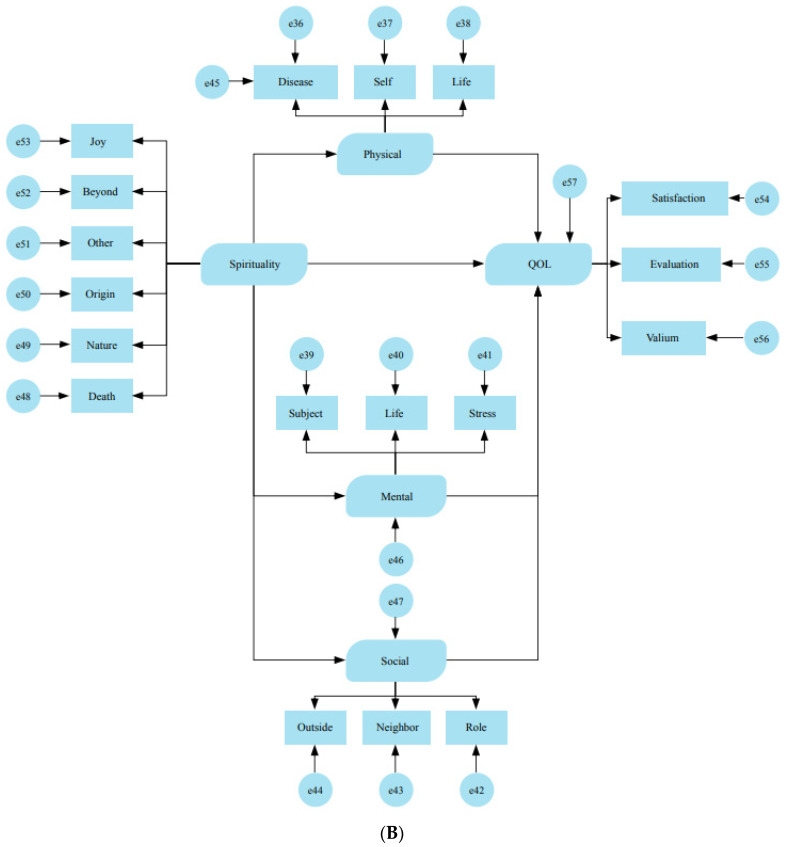
The causal structure model in which spirituality is a distinct item influencing QoL (Model 1, (**A**)) and a causal structure model in which spirituality poses direct and indirect effects on QoL (Model 2, (**B**)). The number assigned to each variable refers to the order of the corresponding questions in the survey.

The baseline characteristics were summarized in mean, standard deviation, and range for continuous variables and event and frequency for categorical ones. The analysis was conducted on a causal structure model in which spirituality is a distinct item influencing QoL (Model 1, Figure 2A) and a causal structure model in which spirituality poses direct and indirect effects on QoL (Model 2, Figure 2B). 

Next, we conducted additional analysis on the causal structure model (i.e., Model 2) to control for confounding factors (family structure and educational background) presented in Model 3. Family structure as a confounding factor was categorized as whether the person lived alone or not, and educational background was categorized as whether the person attended compulsory education or above. Age, sex, and marital status were used as adjustment factors in the upper part of the figure of Model 3. Noteworthy is that the adjusted results are not shown in the figures due to the large number of paths presented in the diagrams. Therefore, the presentation of the values would have been difficult.

The covariance structure analysis was used to test the comparisons in terms of model fit, standardized path coefficients, overall effect, and coefficient of determination. This choice is explained by the ability of the covariance analysis to specify models that include observed variables and their hypothesized relationships, estimate direct, indirect, and total effects among variables, and provide fit indices to explain how each model fits the observed data. For each model, we reported the goodness-of-fit in terms of the goodness-of-fit index (GFI), adjusted goodness-of-fit index (AGFI), comparative fit index (CFI), and root mean square error of approximation (RMSEA). Finally, to compare the models, we used the Akaike information criterion (AIC).

## 3. Results

The subjects who agreed to be interviewed were 338 elderly people (93 males and 245 females). Approximately three-quarters of the respondents were women (245/338, 72.5%). The average age and its standard deviation were 77.2 ± 6.4 years. The most common family structure was living with a spouse (34.3%), followed by living with a spouse and children (30.2%) and living alone (15.4%). The most common final educational level was up to compulsory education (76.0%), up to high school (20.1%), and university or higher (3.8%). The mean height and standard deviation were 157.4 ± 6.1 cm for men and 144.8 ± 5.3 cm for women, and the mean weight and standard deviation were 59.9 ± 8.9 kg for men and 51.8 ± 8.3 kg for women. The body mass index (BMI) was 24.1 ± 3.0 kg/m^2^ for men and 24.7 ± 3.5 kg/m^2^ for women. Systolic blood pressure was 144.0 ± 20.4 mmHg systolic in men and 145.2 ± 20.2 mmHg in women, whereas diastolic pressure was 77.3 ± 11.4 mmHg and 77.9 ± 11.2 mmHg in men and women, respectively (Table 1). 

For Model 1, the results of the various goodness-of-fit indices are presented in Table 2. The Chi-squared value was 250.480, with a probability of 0.000. No significant reduction in the Chi-squared value was expected by the modification indicators: the GFI was 0.924, the AGFI was 0.900, the CFI was 0.844, and the RMSEA was 0.052. The results of the standardized path coefficients are presented in Table 3. The path coefficient for mental health to QoL was 0.588, which was significant (*p* < 0.01). The path coefficient from social health to QoL was 0.253, which was also significant (*p* < 0.05). The overall effect on QoL (direct effect + indirect effect; however, only the direct effect was used in this model) is shown in Table 4. The largest effect was 0.588 for mental health to QoL, followed by 0.401 for physical health to QoL, then 0.253 for social health to QoL, and the smallest effect was 0.116 for spirituality to QoL. The coefficient of determination for QoL, the objective variable of the model, was 0.583. 

As for Model 2, the results of the various goodness-of-fit indices are presented in Table 5. The Chi-squared value was 227.670, with a probability of 0.000. None of the modification indicators could be expected to significantly reduce the Chi-squared value: the GFI was 0.929, the AGFI was 0.906, the CFI was 0.870, and the RMSEA was 0.048, all of which resulted in higher acceptance levels than in Model 1. The results of the standardized path coefficients are presented in Table 6. The path coefficient from spirituality to spiritual health was 0.363, which was significant (*p* < 0.01). The path coefficient from spiritual health to quality of life was 0.622, which was significant (*p* < 0.01). The overall effect (direct + indirect) on QoL is shown in Table 7. The largest effect was 0.622 for spiritual health to QoL, followed by 0.377 for physical health to QoL, 0.278 for spirituality to QoL, and the smallest effect was 0.240 for social health to QoL. When the overall effect of spirituality on QoL was split into direct and indirect effects, they were −0.008 and 0.286, respectively. This means that the indirect effect of spirituality on QoL is much larger than the direct effect. The coefficient of determination for QoL as the objective variable in this model was 0.610, indicating that a higher proportion was explained than in Model 1. 

A comparison of the results of the analysis of Model 1 and Model 2 is presented in Table 8. The AIC was added as an indicator to compare the models. The value of the AIC is 313.67 for Model 2 compared to 330.48 for Model 1. The relative superiority of Model 2 in terms of the coefficient of determination, goodness of fit index, and AIC is shown.

**Figure 3:**
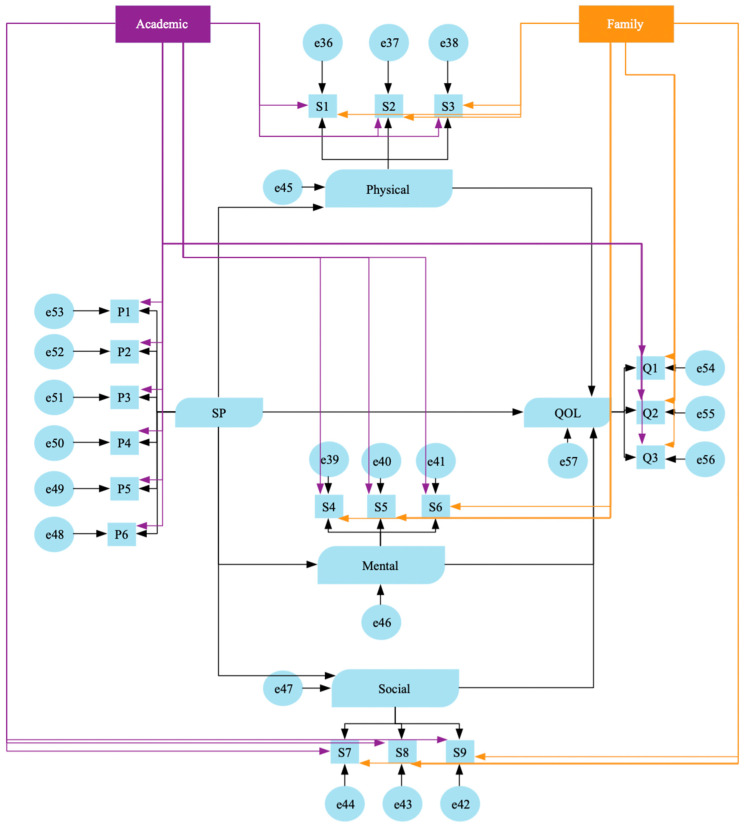
The causal structure model in which spirituality poses direct and indirect effects on QoL after adjusting for family structure and educational background (Model 3).

In the additional analysis model (Figure 3, Model 3), each confounding factor (family structure and educational background) was controlled as an observed variable, and all observed variables in the model had an effect. After controlling for confounding factors, the results of various goodness-of-fit indices were GFI: 0.938, AGFI: 0.899, CFI: 0.890, and RMSEA: 0.046. The fit of the model was found to be almost the same as hypothesis Model 2. Furthermore, in the relationship between each confounder and all observed variables, many standardized path coefficients were not significant. For these reasons, hypothesis Model 2, in which observed variables as confounding factors were excluded from the model, was adopted as the final model.

## 4. Discussion

In this study, we aimed to determine the relationship between spiritual health, mental health, and quality of life in the older population to advocate for amending the definition of health to include spirituality. However, before delving into concrete conclusions, one must consider the wider aspects of the sampled population and how they may differ in a broader context.

Our sample had a BMI of 24.1 ± 3.0 kg/m^2^ for men and 24.7 ± 3.5 kg/m^2^ for women, slightly higher than the mean BMI of 23.1 ± 3.2 kg/m^2^ for men and 23.1 ± 3.6 kg/m^2^ for women aged 70 or older in the 2010 (the time of survey conduction) National Health and Nutrition Survey [29]. Moreover, systolic blood pressure was 144.0 ± 20.4 mmHg in men and 145.2 ± 20.2 mmHg in women, and diastolic blood pressure was 77.3 ± 11.4 mmHg in men and 77.9 ± 11.2 mmHg in women, generally within the normal to borderline hypertension range for the elderly as defined by the WHO in their 1993 guidelines [30]. However, this is regarded as hypertension in more recent global guidelines [31]. Logically, more than half of the participants (206, 60.9%) were taking blood pressure medication. Although this result was slightly higher than the 52.7% result for the use of blood pressure-lowering medication in the National Health and Nutrition Survey [29], the analyzed population in this study was considered to be an average elderly population.

Using quantitative data, the study examined the contribution to QoL of older people regarding a health concept that includes spirituality as a fourth health dimension to physical, mental, and social health. Previous research had proposed that a more comprehensive health model is possible by integrating aspects of physical, mental, and social health from a spiritual perspective and that the three categories of human existence are physical/material (corporeal), mental, and spiritual (spiritual) [32]. Moreover, previous research highlighted that these three spheres are not to be equated and parallel, but that spirituality occupies a position of foundation on which corporeal and mental can be built [33]. 

In our study, it was found that spirituality did not exert a direct effect on the QoL of older people, but an indirect effect via social and mental health, with a particularly strong contribution to mental health. This effect could be attributed to spirituality’s contribution to increasing individuals’ resilience, providing a sense of purpose, and meaning of life, promoting positive relationships with others, offering hope and optimism, and helping individuals cultivate gratitude and a sense of self-understanding. This is in line with Al-Natour et al. and Chaar et al., who observed an indirect positive correlation between spirituality and QoL in cancer patients mediated by social, functional, physical, and mental factors [34]. Contrastingly, an integrative review of the research conducted on spirituality in the past decade revealed that, in addition to an indirect effect, spirituality exerted a direct effect on QoL. Additionally, a negative association was also reported between spirituality and certain aspects of QoL such as sexual health and sleep behavior, with patients with stronger spiritual health being more prone to develop insomnia [35]. 

As physical health was hardly involved in this (standardized path coefficient from spirituality to physical health: 0.048), it was considered appropriate to view it as an antagonistic relationship with spirituality. In other words, it was thought that this may be an indication that spirituality is stronger in the physically frail, which has been previously reported [36,37]. This can be easily imagined as older people are likely to be in a crisis where they are forced to reconsider the meaning of their existence due to loss of physical functions, bereavement of family and friends, retirement from social roles, and so on. Hence, they tend to view their death as an inevitable end, more realistically and routinely, and, as with terminally ill cancer patients, the elderly living with old age can be seen as a population with a growing interest in spirituality. 

We believe that future cohort studies will demonstrate that even if physical health declines, it is possible to maintain or improve QoL by increasing spirituality and influencing mental health. Regarding the fact that there was little direct effect between spirituality and QoL (standardized path coefficient from spirituality to QoL: −0.008), the results of a meta-analysis of studies examining the relationship between spirituality and QoL stated that although the relationship between the two was not completely unrelated, it was not necessarily strong. This may be due to the existence of some mediating factor between the two relationships [38]. In this study, we hypothesized the mediating factor to be precisely mental health. 

### Limitations

This study has some limitations, some of which were previously discussed in various parts. These include the slightly different characteristics of the sample when compared to the average Japanese population. Moreover, it was not feasible to include more people due to the nature of the data collection (interviews). Additionally, females were more likely to respond to our call for interviews and were more engaging, rendering the results of this study more representative of the female population. Although covariance structure analysis offers a potent framework for analyzing intricate causal linkages, such models are usually better at estimating association rather than causality. Therefore, caution should be exerted when extrapolating causation from these models alone and multiple sources of evidence should be consulted to enhance the strength of causal interpretations. Similarly, the choice of a survey-based cross-sectional study challenges the establishment of causality due to the lack of temporal sequence which hinders the ability to determine whether the exposure preceded the outcome and cannot highlight the presence of reverse causation. Moreover, cross-sectional studies are more vulnerable to confounding, have less control over external factors that might affect the outcome, and are susceptible to recall bias. Another important factor to take into consideration is that spirituality may be influenced by ancestral folk beliefs and that these beliefs in Kumejima town are almost identical. Therefore, future studies need to consider the effect of different religions (as well as atheism) on spirituality and QoL. Additionally, our scale was developed, tested, and implemented in Japanese in a previous study [27]. Therefore, further research would require proper psychometric validation and translation if used in a global setting [39]. Lastly, conducting this survey in urban areas may yield very different results.

## 5. Conclusions

The study findings underscore the interplay between spiritual health, mental health, and QoL among older individuals, suggesting a need to broaden the understanding of QoL beyond mere physical health. While our research provides valuable insights into this association, we acknowledge the complexity of QoL as a multidimensional construct, particularly in the context of aging populations. Moreover, we need to consider the hypothesis of a “compensation logic” in the case of older individuals, wherein factors such as spirituality and mental well-being may mitigate declines in physical health and contribute to overall QoL enhancement. This hypothesis warrants further exploration through deeper research and discussion to unravel the intricate dynamics at play. Furthermore, longitudinal research designs will be instrumental in uncovering the evolving trajectories of QoL among older adults, elucidating how changes in spirituality and mental health intersect with shifts in physical health status and subjective well-being over time. By embracing the complexity of QoL and incorporating spirituality as a crucial component, we can enrich interventions aimed at enhancing the well-being of older individuals and foster a more holistic approach to aging and health.

## Figures and Tables

**Table 1:** Baseline characteristics of the sample (*n* = 338). Continuous variables are presented in mean ± standard deviation (range).

Category		Frequency	(%)
Sex	Male	93	27.5%
	Female	245	72.5%
Age (years)		77.2 ± 6.4 (65–94)	
Family structure	Single	52	15.4%
	Married	116	34.3%
	Married with children	102	30.2%
	Separate with children	56	16.6%
	Other	12	3.6%
Education	Below high school	257	76.0%
	High school	68	20.1%
	University or above	13	3.8%
Height (cm)	Actual value	148.3 ± 7.9 (129.5–174.0)	
	Self-reported value	149.4 ± 8.1 (120.0–175.0)	
Weight (Kg)	Actual value	54.0 ± 9.2 (31.5–83.6)	
	Self-reported Value	53.9 ± 8.7 (32.0–78.0)	
BMI (kg/m^2^)	Actual values	24.5 ± 3.4 (15.3–35.7)	
	Self-reported value	23.9 ± 3.1 (16.0–34.2)	
Blood pressure (mmHg)	Systolic blood pressure	144.9 ± 20.3 (87.0–216.0)	
	Diastolic blood pressure	77.8 ± 11.2 (42.0–115.0)	
Taking medication for blood pressure	Yes	206	60.9%
	No	132	39.1%

**Table 2:** Main goodness-of-fit indicators for hypothesis Model 1.

Fit Indicators		*p*-Value
Chi-squared test	Chi-squared value	250.480
	Degree of freedom	131
	Probability	0.000
GFI		0.924
AGFI		0.900
CFI		0.844
RMSEA		0.052

Abbreviations: GFI (Goodness-of-fit index); AGFI (Adjusted goodness-of-fit index); CFI (Comparative fit index); RMSEA (Root mean square error of approximation).

**Table 3:** Results of covariance structure analysis for hypothesis Model 1 (standardized path coefficients).

Path		Path Factor	Significant Difference
Physical health	QoL	0.401	-
Mental health		0.588	**
Social health		0.253	*
Spirituality		0.116	-
Physical health	Disease	0.176	-
	Self-awareness	0.676	-
	Life activities	0.113	※1
Mental health	Subjectivity	0.638	※1
	Life satisfaction	0.569	**
	Stress	0.441	**
Social health	Role in society	0.423	※1
	Neighborhood	0.502	**
	Going outside	0.467	**
Spirituality	Dying	0.233	**
	Nature	0.623	**
	Origin	0.715	**
	Other	0.638	**
	Go above and beyond yourself	0.554	**
	Joy of living	0.691	**
QoL	Satisfaction	0.469	※1
	Evaluation	0.387	**
	Valium	0.533	**

※1 The path to the observed variable is fixed to 1. ** *p* < 0.01. * *p* < 0.05.

**Table 4:** Effects of the dependent variable for hypothesis Model 1 (direct and total effects).

Dependent Variable	Independent Variables	Direct Effects	Indirect Effects	Overall Effects
QoL	Physical health	0.401	-	0.401
	Mental health	0.588	-	0.588
	Social Health	0.253	-	0.253
	Spirituality	0.116	-	0.116

**Table 5:** Main goodness-of-fit indicators for hypothesis Model 2.

Fit Indicators		*p*-Value
Chi-squared test	Chi-squared value	227.670
	Degree of freedom	128
	Probability	0.000
GFI		0.929
AGFI		0.906
CFI		0.870
RMSEA		0.048 *

Abbreviations: GFI (Goodness-of-fit index); AGFI (Adjusted goodness-of-fit index); CFI (Comparative fit index); RMSEA (Root mean square error of approximation); * *p* < 0.01.

**Table 6:** Results of covariance structure analysis for hypothesis Model 2 (standardized path coefficients).

Path		Path Factor	Significant Difference
Spirituality	Physical health	0.048	-
	Mental health	0.363	**
	Social Health	0.178	0.065
Physical health	QoL	0.377	-
Mental health		0.622	**
Social Health		0.240	0.054
Spirituality		−0.008	-
Physical health	Disease	0.182	-
	Self-awareness	0.664	-
	Life activities	0.113	※1
Mental health	Subjectivity	0.587	※1
	Life satisfaction	0.630	**
	Stress	0.419	**
Social health	Role	0.403	※1
	Neighborhood	0.541	**
	Go outside	0.445	**
Spirituality	Dying	0.235	**
	Nature	0.613	**
	Origin	0.719	**
	The Other	0.633	**
	Go above and beyond yourself	0.549	**
	Joy of living	0.700	**
QOL	Satisfaction	0.503	※1
	Evaluation	0.377	**
	Valium	0.521	**

※1 The path to the observed variable is fixed to 1. ** *p* < 0.01.

**Table 7:** Effects on the dependent variable for hypothesis Model 2 (direct, indirect, and total effects).

Dependent Variable	Independent Variables	Direct Effects	Indirect Effects	Overall Effects
Physical health	Spirituality	0.048	-	0.178
Mental health	Spirituality	0.363	-	0.363
Social Health	Spirituality	0.178	-	0.048
QoL	Physical health	0.377	-	0.377
	Mental health	0.622	-	0.622
	Social Health	0.240	-	0.240
	Spirituality	−0.008	0.286	0.278

**Table 8:** Comparison of covariance structure analysis results in hypothesis Models 1 and 2 (main goodness-of-fit indicators and coefficients of determination of dependent variable).

Model	Chi-Squared Test			GFI	AGFI	CFI	RMSEA	AIC	Coefficient of Determination (QoL)
	Chi-Squared Value	Degree of Freedom	Probability						
Hypothesis Model 1	250.480	131	0.000	0.924	0.900	0.844	0.052	330.480	0.583
Hypothesis Model 2	227.670	128	0.000	0.929	0.906	0.870	0.048	313.670	0.610

Abbreviations: GFI (Goodness-of-fit index); AGFI (Adjusted goodness-of-fit index); CFI (Comparative fit index); RMSEA (Root mean square error of approximation); AIC (Akaike information criterion).

## Data Availability

All data used in this study can be obtained from the first author upon a reasonable request.

## Supplementary Materials

The following supporting information can be downloaded at: https://www.mdpi.com/article/10.3390/ejihpe14030050/s1, Table S1: CROSS Checklist—Checklist of items that should be included in reports of survey studies.

## Abbreviations

AGFI	Adjusted goodness-of-fit index
AIC	Akaike information criterion
BMI	Body mass index
CFI	Comparative fit index
CROSS	Consensus-Based Checklist for Reporting of Survey Studies
GFI	Goodness-of-fit index
QoL	Quality of life
RMSEA	Root mean square error of approximation
SP Health Scale	Spirituality Health Scale for the Elderly
WHO	World Health Organization
